# Asian Americans & Racism: Individual and Structural Experiences (ARISE) Pilot Study: Cultural and Linguistic Considerations in Studying Chinese, Vietnamese, and Korean American Older Adults

**DOI:** 10.1002/alz70857_107405

**Published:** 2025-12-26

**Authors:** Annie Ching, Anson Wong, Bora Nam, Marian Tzuang, Linh Nguyen, Seoyeon Yum, Jessie Deng, Daren Huang, Oanh L. Meyer, Josh D Grill, Haeok Lee, Boon Lead Tee, Quyen Q Tiet, Quyen Vuong, Hongru Meng, Bobin Kim, Luu Le, Nhu Pham, Daisy Nguyen, Tien Nguyen, Van Ta Park

**Affiliations:** ^1^ University of California San Francisco School of Nursing, San Francisco, CA, USA; ^2^ Memory and Aging Center, Department of Neurology, University of California San Francisco, San Francisco, CA, USA; ^3^ University of California, Davis School of Medicine, Sacramento, CA, USA; ^4^ University of California, Irvine, Irvine, CA, USA; ^5^ Meyers College of Nursing, New York University, New York, NY, USA; ^6^ California School of Professional Psychology at Alliant International University, San Francisco, CA, USA; ^7^ International Children Assistance Network, Milpitas, CA, USA; ^8^ University of California, Davis, Davis, CA, USA; ^9^ School of Nursing, Department of Community Health Systems, University of California, San Francisco, CA, USA

## Abstract

**Background:**

Research has shown that individual and structural discrimination are linked to risk of Alzheimer's disease and related dementias (ADRD). This link remains understudied in Asian Americans, despite the rise in anti‐Asian hate stemming from the COVID‐19 pandemic. The Asian Americans and Racism: Individual and Structural Experiences (ARISE) pilot study aims to explore associations of discrimination experiences to ADRD risk while evaluating the feasibility of recruiting Chinese‐, Korean‐, and Vietnamese‐speaking older adults (≥65 years old) for cognitive testing and blood draw. This abstract highlights cultural and linguistic considerations when working with these populations in ADRD research.

**Method:**

Assessments are conducted in participants’ primary language (Cantonese, Korean, Mandarin, Vietnamese) and include: 1) a blood draw to test cardiometabolic and ADRD biomarkers; and 2) an interview that includes cognitive testing, anthropometric and vital signs measurements, sociodemographic background, medical history, mental health, social support, and multi‐level discrimination experiences spanning from everyday and major lifetime experiences to residential segregation and structural anti‐immigrant prejudice. Validated assessments in each language were sourced by a language committee. If unavailable, instruments were translated and adapted following the World Health Organization translation guidelines. Examiners undergo training with cognitive experts in each language to ensure culturally appropriate assessments.

**Result:**

Recruitment strategies are tailored in collaboration with community organizations to align with each language group's preferences, including adjusting the blood draw and interview sequence and hosting group blood draws where participants completed the procedure together, fostering a sense of community and reducing anxiety. Engaging in outreach events and providing culturally relevant incentives (i.e. Asian grocery store gift cards) helped foster trust and encourage retention. As of January 23, 2025, 252 participants have enrolled (112 Vietnamese, 56 Korean, 84 Chinese), with 217 completing both blood draw and interview, and the remaining participants awaiting scheduling.

**Conclusion:**

The ARISE pilot study demonstrates that collaborations with community partners played an important role in facilitating successful recruitment and study completion, specifically in conducting cognitive assessments and collecting blood samples with older Asian Americans in ADRD research. Future findings will be crucial in guiding future ADRD research in underrepresented populations.

